# Polishing of EB-PBF Ti6Al4V Vertical Surfaces with Semi-Melted Particle Characteristics Realized by Continuous Laser

**DOI:** 10.3390/mi17010046

**Published:** 2025-12-30

**Authors:** Xiaozhu Chen, Congyi Wu, Youmin Rong, Guojun Zhang, Yu Huang

**Affiliations:** 1 State Key Lab of Digital Manufacturing Equipment and Technology, Huazhong University of Science and Technology, Wuhan 430074, China; 2 School of Mechanical Science and Engineering, Huazhong University of Science and Technology, Wuhan 430074, China

**Keywords:** laser polishing, EB-PBF Ti6Al4V, vertical surface, molten pool dynamics

## Abstract

Electron beam powder bed fusion (EB-PBF) Ti6Al4V often exhibits high vertical surface roughness, limiting its use in high-end applications. In this study, an infrared continuous-wave laser was applied to precisely polish the vertical surface. An orthogonal design identified the optimal condition as 10,400 kW/cm^2^ power density, 800 mm/s scanning speed, and one pass, achieving a minimum Sa of 0.24 μm and a 98.03% reduction compared with the as-built surface. To address the adhered semi-molten particle characteristics of EB-PBF sidewalls, a molten-pool-dynamics-based polishing model was developed and validated, yielding an error as low as 1.24%. Simulations indicate that power density governs the final morphology by controlling molten pool coverage, scanning speed affects polishing efficiency via thermocapillary force, and polishing time influences surface quality by triggering or avoiding melt splashing.

## 1. Introduction

**Figure 1:**
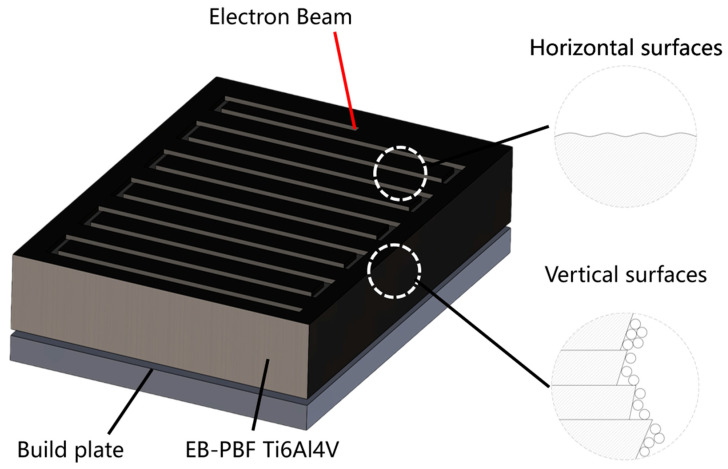
Schematic of surfaces in EB-PBF parts.

Ti6Al4V is widely used in automotive, energy, chemical, and biomedical industries [1,2,3] due to its excellent physicochemical properties, such as high strength, low density, corrosion resistance, and biocompatibility [4,5,6,7]. However, for the processing and fabrication of Ti6Al4V products, the traditional machining and fabrication processes can hardly avoid a large amount of material waste and long lead times [8,9]. Electron beam powder bed fusion (EB-PBF), as an advanced manufacturing process for producing 3D parts layer-by-layer based on digital models [10,11,12], can effectively solve the above problems of Ti6Al4V materials and provide them with the ability to produce parts with high geometric complexity [11,13]. Parts produced by the EB-PBF process can be classified into horizontal and vertical surfaces based on their relative orientation to the build plate [14]. The vertical surface is the interface between the molten and unmelted zones in the EB-PBF process, so there is always insufficient energy to melt the metal powder [15]. The high-roughness characteristics caused by the hemispherical shape of the incompletely melted particles adhering to the vertical surface are one of the factors influencing the EB-PBF Ti6Al4V parts to expand into the aerospace field [16]. The surface characteristics of the EB-PBF parts are shown in Figure 1.

Laser polishing is a technique that uses high-intensity lasers to remelt the surface of a part, reducing surface roughness and improving the microstructural quality of the part to some extent. It has become one of the most promising post-processing methods for metal additive manufacturing parts. Current research on laser polishing is mainly divided into pulsed-laser polishing with ablation as the mechanism and continuous-laser polishing with remelting as the mechanism [17,18,19,20]. For the study of pulsed-laser polishing, Debajyoti and co-workers [21] polished SLM AlSi10Mg parts using a nanosecond laser, reducing the surface roughness by 80–88%, and comprehensively evaluated the residual stress, microhardness, and microstructure of SLM AlSi10Mg samples laser-polished in air and argon atmospheres. Yuan et al. [22] investigated the effect of femtosecond laser incidence angle on the polishing performance of C/SiC ceramic matrix composites. Their results showed that, at a single-pulse energy of 71.53 μJ, a defocus distance of −0.13 mm, an incident angle of 20.61°, and a Y-direction spot overlap ratio of 77.50%, an optimal surface roughness of Ra = 1.03 μm was achieved. Chen et al. [23] used a picosecond laser to polish the surface of ASP23 steel and achieved a small heat-affected zone (8 μm) while reducing the surface roughness of the part from 0.22 μm to 0.159 μm. Based on the above studies, it can be seen that pulsed-laser polishing of metallic materials is mainly limited to the surface of parts with low initial roughness (Sa < 5 μm), and it is difficult to eliminate the peaks and valleys by ablation for parts with high roughness. Therefore, continuous-laser polishing is often used for high-roughness part surfaces. Xu and co-workers [24] polished 304 stainless steel using a continuous fiber laser. The experimental results showed that the surface roughness of the polished area decreased from 2.109 μm to 1.114 μm. Wang et al. [25] used a continuous laser to polish the surface of laser powder bed fusion Inconel 718 parts with the assistance of a magnetic field, and the surface roughness was reduced from 6.51 μm to 0.13 μm, while the presence of a magnetic field suppressed the microscopic segregation of alloying elements. The use of a continuous-laser polishing process to improve the high-roughness characteristics of the surface of EB-PBF Ti6Al4V parts is a well-established method, but there are fewer studies on the optimization of laser polishing process parameters for the high-roughness vertical surface of EB-PBF Ti6Al4V parts with hemispherical-shape unmelted-powder characteristics.

To study the mechanism of laser polishing, Li et al. [26] established a two-dimensional axisymmetric model to describe the melt pool dynamics and surface morphology evolution during laser polishing. Ma et al. [27] developed a two-dimensional transient thermal-fluid coupling model for nanosecond laser polishing, which revealed the influence of pulse width on the molten pool shape. Zhang and co-workers [28] developed a two-dimensional cross-sectional model for continuous-wave laser polishing that simultaneously accounts for heat transfer, fluid flow, and material vaporization, and used it to analyze the formation mechanism of the “M”-shaped surface structure. Wang et al. [29] introduced a pulsed-laser equivalent finite-element model that considers thermal radiation, thermal convection, and thermal conduction in laser processing, which achieved accurate simulation of processing contours with an error of less than 9% compared to experimental results. However, there has been no study on the laser polishing mechanism considering the vertical surface morphology of EB-PBF Ti6Al4V parts, while the molten pool dynamics mechanism of the laser polishing process has not been well revealed.

An infrared continuous-wave laser was employed to polish EB-PBF Ti6Al4V vertical surfaces. Orthogonal experiments reduced Sa from 13.18 μm to 0.26 μm at the optimal condition (LPPD = 10,400 kW/cm^2^, LPS = 800 mm/s, one pass). To capture the adhered semi-molten particles on EB-PBF sidewalls, a molten-pool-dynamics-based polishing model was developed and validated, achieving a minimum error of 1.24%. Simulations further show that LPPD governs the final morphology by controlling molten pool coverage, LPS acts mainly through thermocapillary force, and excessive polishing time can induce melt splashing and degrade surface quality.

## 2. Methods

### 2.1. Experimental Methods

#### 2.1.1. Sample Preparation

Based on the process reported by Peng [20], Ti6Al4V metal powder with a particle size of 53–105 μm was used as the raw material, and its chemical composition is shown in Table 1. The EB-PBF Ti6Al4V samples were prepared using an electron beam selective melting device (Y-150, SAILONG AM Co. Ltd., Xi’an, China), and the processing was started after preheating the EB-PBF building plate to 760 °C. The electron beam was operated at a high voltage of 60 kV, a current of 14.6 mA, a scanning speed of 5800 mm/s, and a scanning pitch of 100 μm, and the process was performed in a vacuum of 5 × 10^−3^ Pa. Finally, the samples were ultrasonically cleaned in anhydrous ethanol for 20 min and blown dry with compressed air.

#### 2.1.2. Laser Polishing Experimental Process

**Figure 2:**
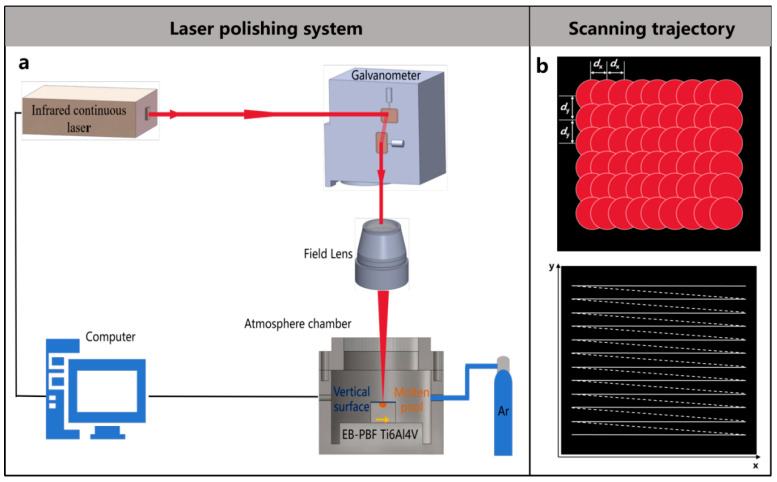
(**a**) Schematic diagram of the laser polishing experimental system (**b**) Laser scanning trajectory.

The experimental laser polishing system used in this study is shown in Figure 2a. The system consists of a laser processing section, a control system, and an atmospheric chamber. The laser processing section uses a fiber laser (YLR-450, IPG PHOTONICS, Oxford, MA, USA) with a laser wavelength of 1070 nm, a maximum power of 450 W. A scanning galvanometer was used to control the scanning trajectory of the laser beam, which is schematically shown in a Z-shape in Figure 2b. At the same time, the laser beam is focused on the moving platform through the field lens, with a focal length of 245 mm. High-purity argon gas with a mass fraction of 99.99% is used as the shielding gas at a flow rate of 10 L/min to ensure that the sample does not oxidize during the LP process.

**Figure 3:**
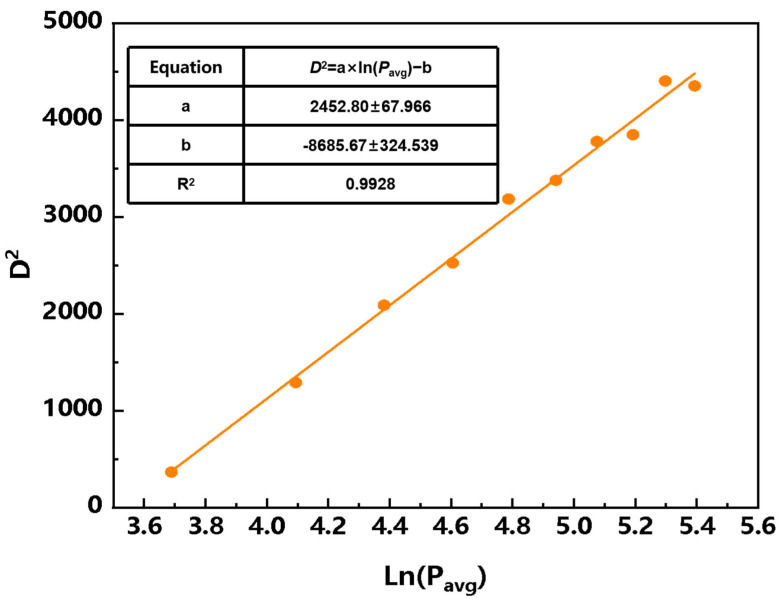
Functional relationship between the square of the ablation-hole diameter *D*^2^ and the logarithm of the average laser power Ln(*P*_avg_).

A laser spot-drilling experiment was conducted, and the spot diameter of the laser used in this study was measured by the area extension method [30]. The irradiation time for each spot was 1 s, and the laser power was set to 40, 60, 80, 100, 120, 140, 160, 180, 200, and 220 W. According to previous studies [31,32,33], the square of the ablation-hole diameter exhibits a functional relationship with the logarithm of the average laser power, as expressed in Equation (1). Figure 3 shows the variation in the square of the ablated-region diameter D2 with the logarithm of the single-pulse energy. From the slope of the fitted line, the laser spot diameter used in this study was determined to be 35 μm.

(1) $$D^{2}=2\omega^{2}\ln(P_{\mathrm{avg}})-2\omega^{2}\ln(\frac{2t}{\pi F_{\mathrm{th}}\omega_{0}^{2}})$$

Prior to laser polishing, the initial EB-PBF Ti6Al4V samples obtained in Section 2.1.1 were cut into 20 mm × 20 mm × 20 mm specimens by wire cutting. The samples were ultrasonically cleaned in acetone to avoid the effect of impurities such as oil on laser polishing. Based on preliminary experiments, laser polishing power density (LPPD), laser polishing speed (LPS), and laser polishing times (LPT) are essential factors that affect the polishing effect of laser polishing on additive manufacturing materials. Therefore, in this study, a three-factor, four-level orthogonal experiment was designed to evaluate the effect of these process parameters on the quality of laser polishing. The levels and values of these process parameters are shown in Table 2, and the specific process parameters for each experiment are shown in Table 3.

The initial morphology and the morphology after laser polishing of the samples were observed using a scanning electron microscopy system (SEM, SU3900, HITACHI, Tokyo, Japan) and a laser scanning confocal microscope (LSCM, VK-X200K, KEYENCE, Osaka, Japan). The roughness of the samples before and after laser polishing was measured using a white light interferometer (WLI, SNEOX, SENSOFAR, Terrassa, Spain).

### 2.2. Simulation Methods

#### 2.2.1. Assumptions and Control Equations

**Figure 4:**
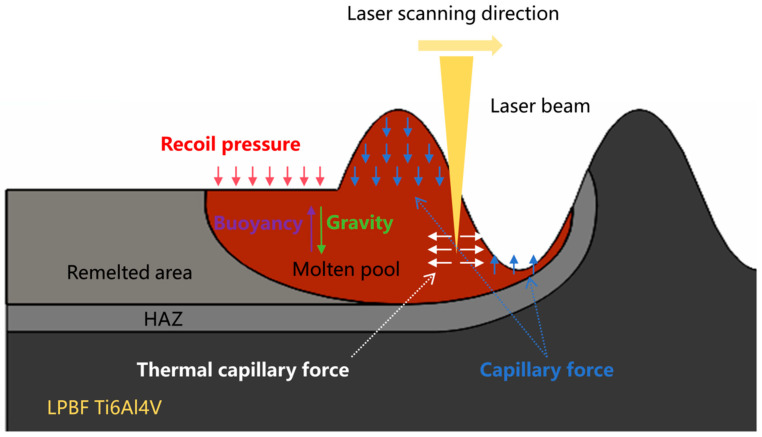
The physical model of the laser polishing process.

The physical model of the laser polishing process is shown in Figure 4. The process can be described as a laser beam of a certain power and wavelength irradiating the surface of the additive manufacturing material with high roughness at a certain scanning speed. As the material surface absorbs the laser energy, the material gradually melts to form a molten pool. Under the combined action of surface and volumetric forces, the molten pool undergoes fluid flow, redistributing the liquid material and reducing the surface roughness of the additive manufacturing parts to achieve a smooth surface. Therefore, based on the above physical phenomena, this study established a continuous-laser polishing model for EB-PBF Ti6Al4V. The physical property parameters of the material are listed in Table 4. The following reasonable simplifications and assumptions were made to save computational resources while ensuring calculation accuracy:

(1) Assume that the fluid in the laser polishing melt pool is an incompressible Newtonian fluid and that the flow state is laminar. The influence of the argon atmosphere on the molten pool is neglected.
(2) Assume that the material is isotropic and that the variation in the material’s physical properties is only related to temperature.
(3) Disregard the effect of melt spatter and plasma during laser polishing.

The numerical calculations in the laser polishing process are described by the following set of governing equations: mass conservation Equation (2) [34], momentum conservation Equation (3) [35], and energy conservation Equation (4) [36].

(2) $$\rho \nabla \cdot \vec{V}=0$$

(3) $$\rho \frac{\partial \vec{V}}{\partial t}+\rho \cdot (\nabla \vec{V})\vec{\cdot V}=\nabla \cdot [-pI+\mu \nabla \vec{V}+\mu \cdot {\left(\nabla \vec{V}\right)}^{T}]+{\vec{F}}_{D}+\rho g\cdot (1-\beta )\cdot (T-T_{m})+F_{M}$$

(4) $$\rho {C_{p}}^{*}\frac{\partial T}{\partial t}+\rho {C_{p}}^{*}\nabla \cdot (\vec{V}T)=\nabla \cdot (k\nabla T)+Q_{M}$$

where ρ is the density of Ti6Al4V, ∇ is the Hamiltonian operator, $\vec{V}$ is the velocity vector, *t* is the time, *I* is the identity matrix, μ is the dynamic viscosity of Ti6Al4V, *T* is the material temperature, ${\vec{F}}_{D}$ is the Darcy force, *g* is the gravitational acceleration, β is the thermal expansion coefficient of Ti6Al4V, *F*_M_ is the source term in the momentum equation, $C_{p}^{*}$ is the equivalent specific heat capacity of Ti6Al4V, *k* is the thermal conductivity of Ti6Al4V, and *Q*_M_ is the source term in the energy equation.

The melting and solidification behavior of the laser polishing process occurs simultaneously, and the position of the melt pool changes with time, making it difficult to distinguish between solid and liquid domains in geometric modeling. In this model, all regions are set as fluids, and the solid and liquid phases in the laser polishing process are distinguished by introducing the Kozeny–Carman Equation (5) of the Darcy force and the Liquid Fraction Function (6), while the solid–liquid transition boundary of the material is defined by both the solid-phase temperature *T_s_* and the liquid-phase temperature *T_l_* of the material. The Kozeny–Carman equation varies with the liquid fraction, and the paste region constant A_m_ is a large constant, and b is a small constant to prevent the denominator of the function from being zero. Therefore, when the material is completely liquid, the Darcy force is 0, which has no additional effect on the flow of the melt; when the material is completely solid, the Darcy force overwhelms the other terms in the momentum equation by a significant value, resulting in a flow velocity of zero; when the material is in the process of solid–liquid conversion, the Darcy force will affect the transient, convective, and diffusion components in the momentum equation to varying degrees depending on the numerical value of the liquid fraction [22].

(5) $$\vec{F_{D}}=-A_{m}(\frac{{\left(1-f_{l}\right)}^{2}}{f_{l}^{3}+b})\vec{V}$$

(6) $$f_{l}=\left\{\begin{array}{cc} 0 & T\leq T_{s}\\ \frac{T-T_{s}}{T_{l}-T_{s}} & T_{s}\leq T\leq T_{l}\\ 1 & T\geq T_{l} \end{array}\right.$$

(7) $$\mu =\mu_{s}+(\mu_{l}-\mu_{s})*flc2hs(T-T_{m},\Delta T)$$

where $\mu_{s}$ is the solid phase dynamic viscosity of Ti6Al4V, $\mu_{l}$ is the liquid phase dynamic viscosity of Ti6Al4V, *T*_m_ is the melting temperature of Ti6Al4V, and ΔT is the solid–liquid phase transition temperature of Ti6Al4V.

Due to the latent heat of phase transition during the solid–liquid–gas phase change, which significantly affects the accuracy of the simulation, an equivalent enthalpy method is used to represent the change in heat capacity during the material phase transition process, as shown in Equations (8) and (9).

(8) $${C_{p}}^{*}=C_{p}(T)+L\cdot G_{m}$$

(9) $$G_{m}=\frac{e^{\frac{-(T-T_{m}{)}^{2}}{\Delta T^{2}}}}{\sqrt{\pi \Delta T^{2}}}$$

#### 2.2.2. Geometric Model

**Figure 5:**
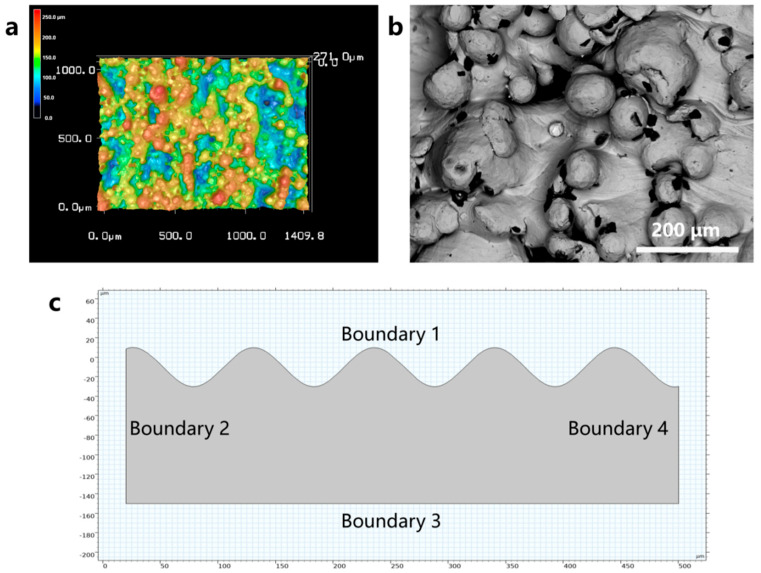
(**a**) LSCM image of the initial morphology of the EB-PBF vertical surface. (**b**) SEM image of the initial morphology of the EB-PBF vertical surface. (**c**) Geometry of the laser polishing numerical simulation model.

Research by Wu and Kamran [23] suggests that incomplete melting of powder adhering to the edge of the molten pool is the primary cause of high roughness in the sidewall morphology of parts produced by powder bed fusion additive manufacturing. Figure 3 shows confocal laser scanning microscopy (LSCM) and scanning electron microscopy (SEM) images of untreated EB-PBF Ti6Al4V samples. The untreated EB-PBF Ti6Al4V sample displays an extremely uneven sidewall surface with significant roughness, as shown in Figure 5a. The main rough feature on the sidewall surface of the EB-PBF Ti6Al4V sample is the adhesion of incompletely melted particles, which appear as hemispherical protrusions ranging in size from tens of microns to over a hundred microns, as shown in Figure 5b. Therefore, this model primarily considers these factors when modeling the rough surface. To ensure computational feasibility, a smoothing treatment is applied to the rough peaks. The geometric characteristics of the model are shown in Figure 5c.

#### 2.2.3. Boundary Conditions

The laser polishing process is described using a Gaussian heat source model to explain the absorption of laser energy. The model also accounts for the effects of thermal convection and radiation on the sample surface.

(10) $$q_{L}=\frac{2\eta Q}{\pi r_{a}^{2}}\exp(-\frac{2r^{2}}{r_{a}^{2}})$$

(11) $$\lambda \frac{\partial T}{\partial \vec{n}}=-h_{c}(T-T_{0})-\varepsilon \sigma (T^{4}-T_{0}^{4})$$

where $q_{L}$ is the heat flux density, η is the laser absorption coefficient of Ti6Al4V, *Q* is the laser power, *r*_a_ is the laser beam radius, $h_{c}$ is the convective heat transfer coefficient, $T_{0}$ is the ambient temperature, ε is the emissivity of Ti6Al4V, σ is the Stefan-Boltzmann constant.

During laser polishing, the material undergoes a solid–liquid–gas phase transition. In laser polishing, the main factors driving the flow of the molten pool are the capillary force generated by the spatial curvature, the thermocapillary force generated by the temperature gradient, and the recoil pressure generated by the evaporation of metal from the surface. The expressions for capillary force, thermocapillary force, and recoil pressure are given in Equations (12)–(14).

(12) $$\sigma_{n}=\kappa \gamma \vec{n}$$

(13) $$\sigma_{t}=\frac{\partial \gamma }{\partial T}\nabla T\cdot \vec{t}$$

(14) $$P_{r}=\frac{1+\beta_{r}}{2}\cdot P_{0}\cdot \exp(\frac{L_{v}m}{K_{B}T_{v}}\cdot (1-\frac{T_{v}}{T}))$$

where $\sigma_{n}$ is the capillary force, κ is the surface curvature, $\vec{n}$ is the surface normal vector, $\sigma_{t}$ is the thermocapillary force, $\frac{\partial \gamma }{\partial T}$ is the surface tension temperature coefficient, ∇T is the temperature gradient, $\vec{t}$ is the surface tangent vector, $P_{r}$ is the metal vapor recoil pressure, $\beta_{r}$ is the reverse diffusion coefficient, $P_{0}$ is the atmospheric pressure, *m* is the atomic mass, $L_{v}$ is the latent heat of vaporization, and $T_{v}$ is the evaporation temperature. The specific application of the boundary conditions in the model can be found in Table 5 and Figure 5c.

This model uses the dynamic mesh method to define the process of surface morphology evolution during polishing. The mesh smoothing method used is Laplace, and the mesh movement equation is shown in Equation (15). The calculation area is divided into triangular meshes consisting of 58,938 units with an average unit mass of 89.03%. The model was solved using COMSOL Multiphysics 6.1.

(15) $$V_{\mathrm{mesh}}\cdot \vec{n}=u_{m}\cdot \vec{n}$$

where $V_{\mathrm{mesh}}$ is the grid movement speed, and $u_{m}$ is the material movement speed.

## 3. Results and Discussion

### 3.1. Orthogonal Experiment

**Figure 6:**
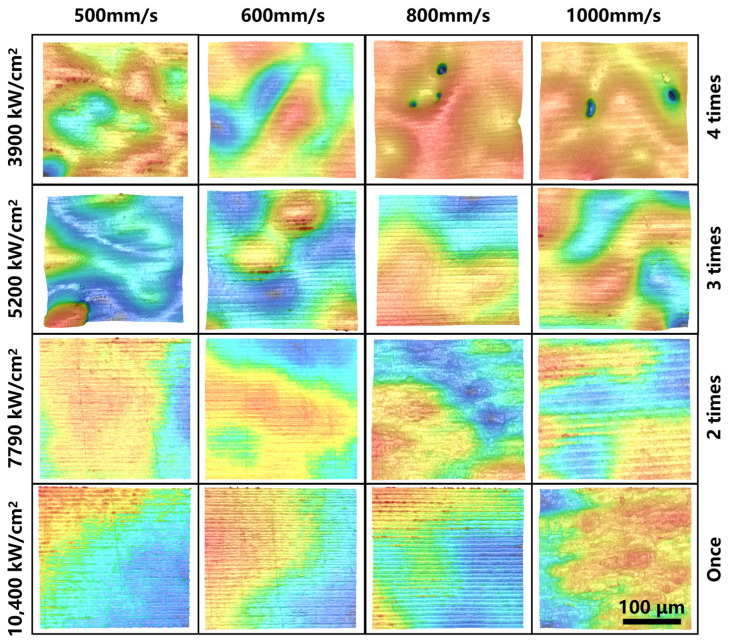
LSCM image of each sample.

The results of the above orthogonal experiments were analyzed by means of variance (ANOVA), which was used to evaluate the effect of controllable factors on the results of the study by decomposing the total variation into different sources of variation and examining the degree of variation between the different groups. The variation of each controllable factor was evaluated by the extreme variance (Range, R), which was used to determine the degree of influence of different controllable factors on the results of the study. The surface roughness, Sa, of the samples after laser polishing was used as an evaluation index of the quality of laser polishing, and it should be emphasized that the average value of three samples was selected for calculating the evaluation index of each polishing process. The statistical data are shown in Table 6, and the typical morphology at each process parameter is shown in Figure 6.

**Figure 7:**
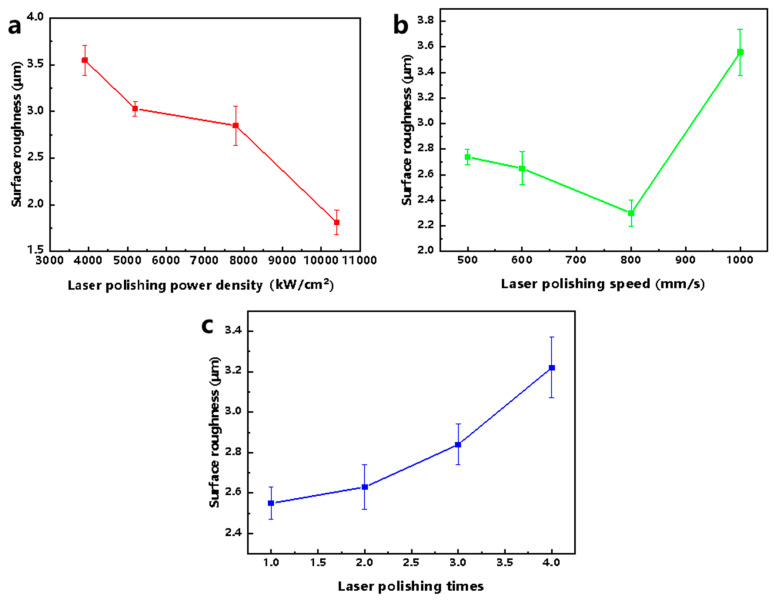
Influence diagram of each process parameter based on surface roughness, Sa, assessment. (**a**) Laser polishing power density. (**b**) Laser polishing speed. (**c**) Laser polishing times.

The main effect analysis can determine the optimal level and the combination of optimal levels in the orthogonal experiments, and the results of the main effect analysis for each process parameter of the orthogonal experiments in this paper are shown in Table 7 and Figure 7. For the process parameter LPP, the range R1 is 1.74 μm, and the optimal level is 4 (10,400 kW/cm^2^); for the process parameter LPS, the range R2 is 1.26, and the optimal level is 3 (800 mm/s); and for the process parameter LPT, the range R3 is 0.67, and the optimal level is 1 (1 time). Thus, the optimum surface roughness Sa can be obtained when the process parameter combination is 4 (10,400 kW/cm^2^)^−3^ (800 mm/s)^−1^ (1 time). Furthermore, by comparing the values of R1, R2, and R3, LPP has the greatest effect on surface roughness, followed by LPS, and LPT has the least effect. Unless otherwise noted, all subsequent studies are conducted using the process parameter combination 4 (10,400 kW/cm^2^)^−3^ (800 mm/s)^−1^ (1 time).

**Figure 8:**
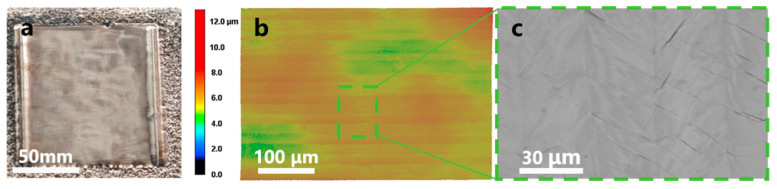
Laser polishing image under optimal process combination. (**a**) Physical image. (**b**) LSCM image. (**c**) SEM image.

Figure 8 shows the surface roughness of EB-PBF Ti6Al4V after laser polishing using the optimal combination of process parameters 4 (10,400 kW/cm^2^)^−3^ (800 mm/s)^−1^ (1). The morphology of EB-PBF Ti6Al4V after laser polishing is shown in Figure 6. The surface roughness, Sa, of the material in the area of 337.82 × 282.62 um measured by WLI is 0.26 μm, and that of the material before polishing is 13.18 μm. The surface roughness of the material has been reduced by 98.03% after laser polishing, and at the same time, the SEM images show that the laser-polished area is homogeneous, and that there are no obvious cracks and defects, such as irregular bumps and depressions.

### 3.2. Laser Polishing Mechanism Based on Molten Pool Dynamics

With the input of laser energy, EB-PBF Ti6Al4V gradually transforms from the solid phase to the liquid and gas phases, and the liquid-phase part of the material flows under surface forces (capillary forces due to spatial curvature difference, thermocapillary forces due to temperature gradient, and recoil pressure due to evaporation) as well as volumetric forces (gravitational and buoyant forces). The distribution of the above three surface forces and laser energy shows a complex non-linear relationship, and the three are coupled in the molten pool flow state during laser polishing over time. Therefore, in order to reveal the influence of process parameters on laser polishing, it is important to investigate the interaction mechanism between laser energy and EB-PBF Ti6Al4V based on molten pool dynamics in conjunction with numerical simulation results.

**Figure 9:**
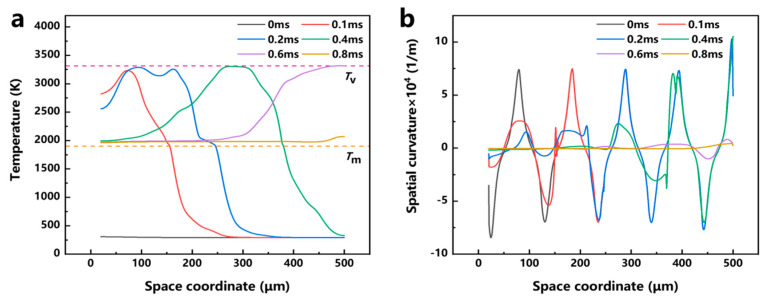
(**a**) Material temperature distribution during laser polishing. (**b**) Spatial curvature distribution during laser polishing.

Figure 9a shows the surface temperature distribution of the material at different moments. It can be seen that the temperature rises sharply when the laser energy is applied to the material. When the processing time reaches 0.1 ms, the highest temperature on the material surface is 3233 K at the center of the laser spot. At this time, the material temperature within the range of nearly 150 μm exceeds the liquidus temperature, forming a molten pool. With time, the temperature gradually increases, and the area exceeding the temperature of the liquid phase line also gradually increases; at the moment of 0.6 ms, the melt pool spreads over the entire material surface, and during the time period of 0.7 ms–0.8 ms, the temperature of the material surface gradually stabilizes at 2000 K due to the movement of the beam, which has already exceeded the processed area. Figure 9b shows the variation of the space curvature of the material at different moments. The value of the space curvature, which describes the degree of curvature of the surface, is used to characterize the roughness of the material morphology, and the larger the space curvature is, the rougher the material surface. As time increases, the space curvature gradually decreases. The space curvature at 0.8 ms is reduced by 95.65% compared to the space curvature at 0 ms, indicating that the roughness of the material has been greatly improved after laser polishing.

**Figure 10:**
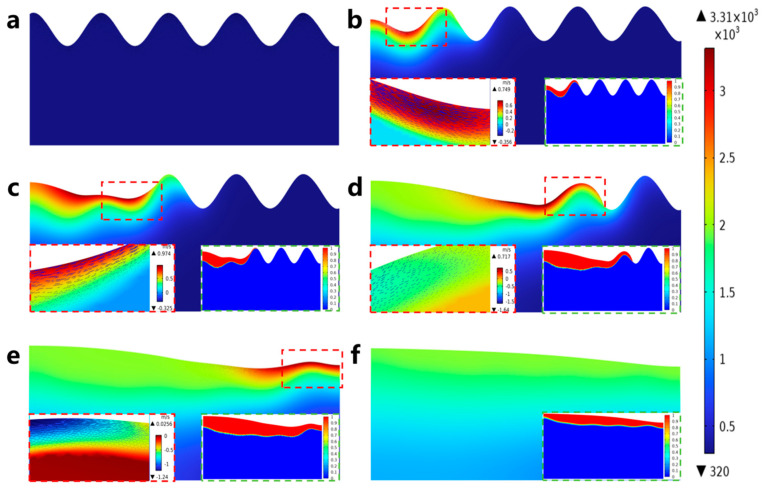
Evolution of laser-polished surface morphology and distribution of phase, temperature, and velocity fields: (**a**) 0 ms, (**b**) 0.1 ms, (**c**) 0.2 ms, (**d**) 0.4 ms, (**e**) 0.6 ms, and (**f**) 0.8 ms.

**Figure 11:**
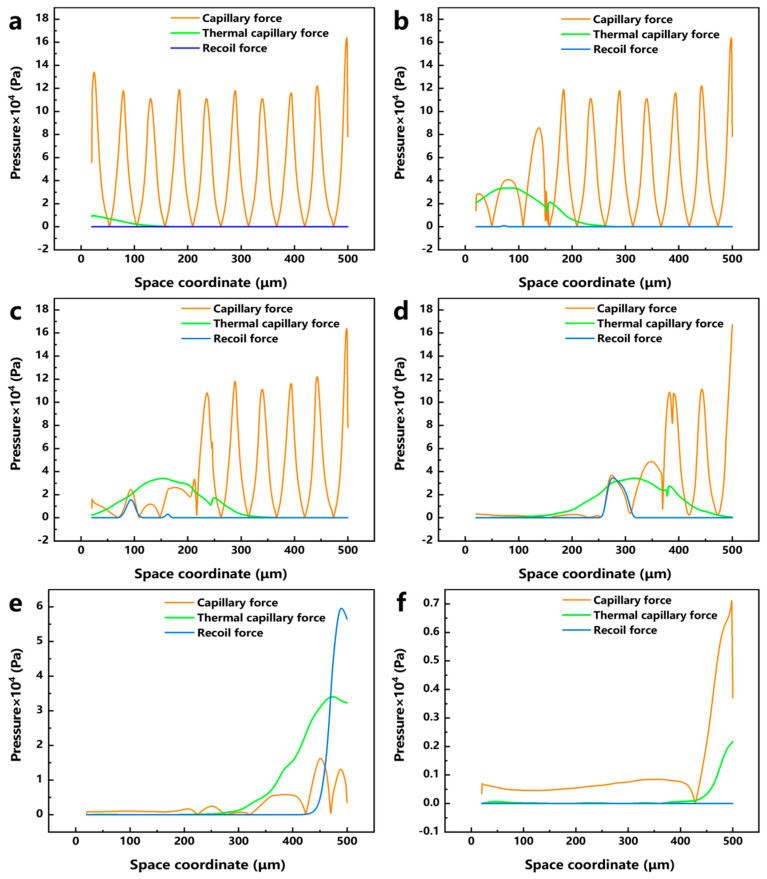
Evolution of capillary force, thermocapillary force, and recoil pressure with time: (**a**) 0 ms, (**b**) 0.1 ms, (**c**) 0.2 ms, (**d**) 0.4 ms, (**e**) 0.6 ms, and (**f**) 0.8 ms.

To further study the motion of the molten pool during laser polishing, numerical simulation data of the temperature field, velocity field, and phase field were visualized, and the dynamics of the molten pool during laser polishing were studied by combining capillary force, thermocapillary force, and recoil pressure maps at different times. Figure 10a–f show the evolution of the surface morphology and phase field distribution at 0, 0.1, 0.2, 0.4, 0.6, and 0.8 ms. The red box displays the velocity field distribution in front of the molten pool, while the green box displays the phase field distribution of the material. In the phase field diagram, the red area represents the liquid phase region and the blue area represents the solid phase region. Figure 11a–f show the evolution of surface forces at 0, 0.1, 0.2, 0.4, 0.6, and 0.8 ms, respectively. From the melt pool evolution process, under the action of laser energy, the convex peaks on the original rough surface gradually disappear under the flow of the melt pool, and the original rough surface becomes smooth.

At the 0 ms moment, because the laser energy just acted on the surface of the material, the highest temperature of 306 K (see Figure 10a) has not yet resulted in a phase transition and has not formed a molten pool; therefore, the material surface morphology has not changed, and, at this time, among the surface forces, the capillary force occupies an absolutely dominant position (see Figure 11a). At the moment of 0.1 ms, the molten pool gradually appears, and the first bump on the rough surface starts to flatten under the action of the surface force (see Figure 10b). In the region of 20 < x < 110 μm, a larger temperature gradient gradually arises with the temperature increase, and the thermocapillary force increases in this region with the same dominant role as that of the capillary force on the flow of the molten pool (see Figure 11b). Since the surface tension of Ti6Al4V due to the negative temperature coefficient of Ti6Al4V, the molten pool moves outward from the center of the laser spot, and the molten material gradually flows from the first peak to the first valley under the action of the thermocapillary force; at this time, the heat generated by the laser has not yet been transferred to other regions, and the surface tension still dominates in the region of 110 < x < 500 μm. At the moment of 0.2 ms, as the temperature continues to rise and the area irradiated by the laser increases (see Figure 10c), the liquid phase region on the material surface grows to 245 μm, and in the 20 < x < 200 μm region, as the phase transition to the liquid phase has already occurred, the spatial curvature becomes flat under the coupling effect of surface forces; the capillary force is gradually reduced, so that the thermocapillary force in this region is gradually dominated (see Figure 11c). At the same time, in the 68 < x < 118 μm region, the maximum temperature reaches 3287 K, which has been close to the evaporation temperature of Ti6Al4V (3315 K). Part of the material in this region is removed by evaporation to produce a recoil pressure. At the moment of 0.2 ms, as the temperature continues to rise and the area irradiated by the laser increases (see Figure 10c), the liquid-phase region on the material surface grows to 245 μm; in the 20 < x < 200 μm region, as the phase transition to the liquid phase has already occurred, the spatial curvature becomes flat under the coupling effect of surface forces. The capillary force is gradually reduced, so that the thermocapillary force of this region gradually dominates (see Figure 11c). At the same time, in the 68 < x < 118 μm region, the maximum temperature reaches 3287 K, which is close to the evaporation temperature of Ti6Al4V (3315 K); part of the material in this region is removed by evaporation to generate a recoil pressure.

**Figure 12:**
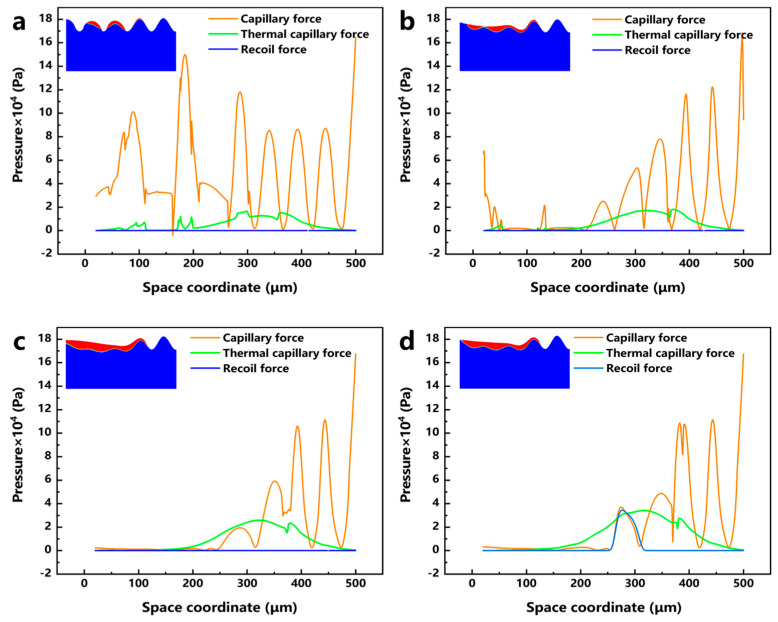
Distribution of capillary force, thermocapillary force, recoil pressure, and corresponding molten pool morphology at different laser polishing power: (**a**) 3900 kW/cm^2^, (**b**) 5200 kW/cm^2^, (**c**) 7790 kW/cm^2^, and (**d**) 10,400 kW/cm^2^.

**Figure 13:**
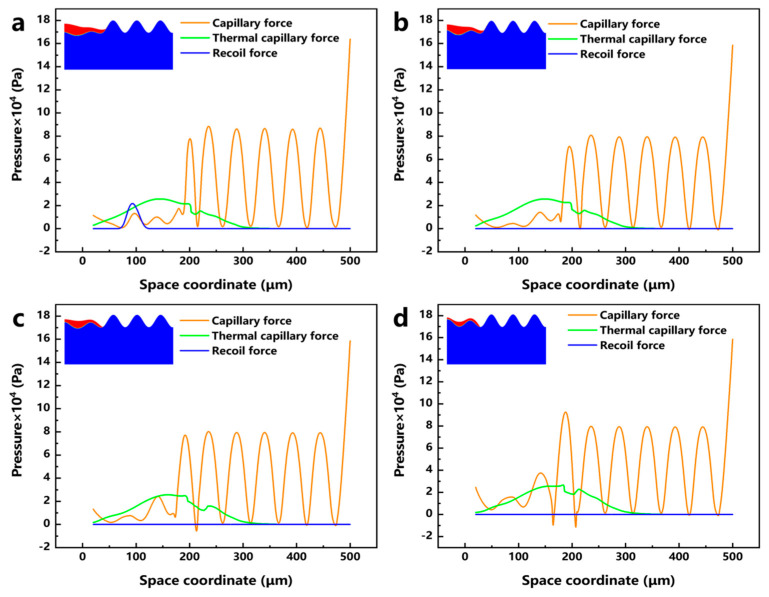
Distribution of capillary force, thermocapillary force, recoil pressure, and corresponding melt pool morphology at different laser polishing speeds: (**a**) 500 mm/s, (**b**) 600 mm/s, (**c**) 800 mm/s, and (**d**) 1000 mm/s.

In order to investigate the influence of different process parameters on the laser polishing effect of EB-PBF Ti6Al4V from the perspective of numerical simulation, the surface force distribution of the molten pool under different LPP and LPS is plotted, and Figure 12a–d shows the surface force distribution and the corresponding molten pool morphology when the LPPD is 3900 kW/cm^2^, 5200 kW/cm^2^, 7790 kW/cm^2^, and 10,400 kW/cm^2^, and the laser polishing time is 0.4 ms, respectively. When the LPPD is 3900 kW/cm^2^, the laser energy is not enough to make the vertical surface of EB-PBF Ti6Al4V entirely covered by the molten pool, which affects the influence of the surface force on the flow and makes the laser polishing effect very poor. When the LPPD is 5200 kW/cm^2^ and 7790 kW/cm^2^, the laser energy is enough to make the molten pool cover the entire vertical surface, but, at this time, the thermocapillary force is always difficult to occupy the dominant position in the occupation of the surface force. According to the previous section, we can see that the appropriate thermocapillary force is the key factor to make the molten pool flow to the two sides, and the lack of thermocapillary force makes ability of the molten pool to move in the tangential direction poor, which affects the final polishing effect. When the LPPD is 10,400 kW/cm^2^, while sufficient laser energy can keep the thermo-capillary force as dominant, at the same time, a reasonable recoil pressure further suppresses the protruding part of the vertical surface. Figure 13a–d shows the surface force distribution and the corresponding melt pool morphology when the LPS is 500 mm/s, 600 mm/s, 800 mm/s, and 1000 mm/s, and the laser focus moves to the same position. When the LPS is 500 mm/s and 600 mm/s, the accumulation of energy makes the thermocapillary force dominant for a long period of time due to the slow laser moving speed. The amount of fluid flowing from the center to the edge is too much, which makes the molten pool show a tilted state and affects the final polishing effect. When the LPS is 1000 mm/s, because the laser energy moves too fast, the accumulated laser energy is not enough to make the vertical surface of the peaks reduce but not completely disappear. When the LPS is 800 mm/s, at this time, the laser power and laser movement speed are in a balanced state, and the vertical surface is in the best flat state. For the effect of LPT, it was experimentally found that when the LPT is more than 1 time, the accumulation of laser energy will induce molten pool spattering, which has a negative impact on the surface roughness of the parts.

This study focuses on the molten pool dynamics of laser polishing and discusses the influence of continuous-laser polishing process parameters on polish quality. The laser energy transforms the material along the polishing path from the solid phase to the liquid and gas phases. After the material becomes liquid, a molten pool is formed. Under the action of surface tension caused by the difference in surface curvature, thermocapillary force induced by a temperature gradient, and vapor recoil pressure during the liquid–gas phase transition, the surface curvature gradually decreases. This eliminates the partially melted particles adhering to the side surface of the sample during the EB-PBF process. At the same time, in the later stages of laser polishing, thermocapillary force and recoil pressure dominate the surface forces due to temperature accumulation. The tangential flow induced by the thermocapillary force and the normal flow induced by the recoil pressure cause the end of the polishing path to flow toward the previously flattened region of the surface curvature, forming smaller waves. The roughness analysis results for the polished side surface of EB-PBF Ti6Al4V show that higher laser polishing power density (LPPD) is advantageous in increasing the coverage area of the melt pool, increasing the surface forces in the melt pool, and improving the leveling ability of the peaks and valleys of the molten pool. A lower LPPD, when faced with a very rough side surface, has difficulty eliminating a large number of semi-melted particles, resulting in poorer polishing effects. Moderate laser polishing speed (LPS) settings help balance the polishing process by preventing excessive laser heat buildup, which could lead to excessive surface forces causing molten pool splashing, or insufficient laser energy, which makes it difficult to eliminate semi-melted particles. In addition, a lower laser polishing time (LPT) is beneficial for slowing heat accumulation under high power conditions.

### 3.3. Experimental Validation of Numerical Simulations

**Figure 14:**
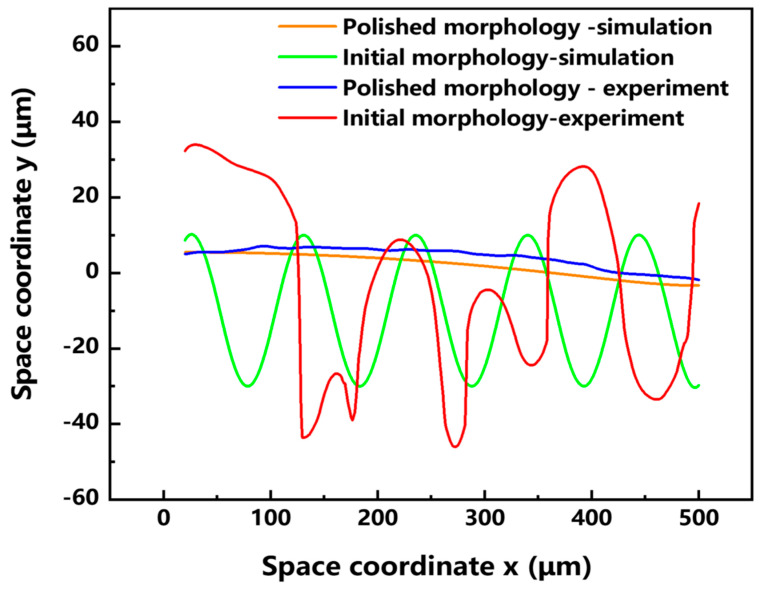
Comparison between numerical model results and experimental results of surface morphology changes in the material before and after polishing.

To verify the accuracy of the numerical model calculation, the surface profile of the material before and after the experiment is measured by LSCM and compared with the results of the numerical model calculation using the same process parameters, as shown in Figure 14, the surface profile of the material after laser polishing becomes smooth, and the difference between the highest and lowest points of the surface profile after laser polishing in the numerical model calculation results is 8.81 μm. In the experimental results, the difference between the highest point and the lowest point of the surface profile curve after laser polishing is 8.92 μm, and the error between the two is 1.24%. The trend of the numerical model calculation and the experimental results is approximately the same. Considering that the numerical calculation model is carried out under a certain amount of assumptions, it can be assumed that the results of the model calculation are more accurate, and it can be used to simulate the laser polishing of EB-PBF Ti6Al4V.

## 4. Conclusions

This study established a melt-pool-dynamics-based laser polishing model for EB-PBF Ti6Al4V vertical surfaces by considering adhered partially molten powders and optimized process parameters via an orthogonal design. The main conclusions are as follows:

(1) Surface roughness decreases with higher laser polishing power density (LPPD) and shorter polishing time (LPT), while extreme scanning speeds (LPS) deteriorate surface quality. The optimum condition (LPPD = 10,400 kW/cm^2^, LPS = 800 mm/s, one pass) achieved Sa = 0.26 μm, corresponding to a 98.03% reduction versus the initial sample surface.
(2) The proposed model agrees well with experiments, with a minimum error of 1.24%.
(3) Simulations indicate that LPPD determines the final morphology by controlling molten pool coverage, LPS regulates polishing efficiency through thermocapillary force, and excessive LPT may induce melt splashing, degrading the surface.

## Figures and Tables

**Table 1:** Chemical composition of Ti4Al4V powders (wt%).

Ti	Al	Fe	Si	V	Y
89.70	6.12	0.021	0.016	4.14	<0.001

**Table 2:** Laser polishing process parameters and their levels.

Manufacturing Parameters	Unit	Notation	Factor Levels			
			1	2	3	4
Laser polishing power density	kW/cm^2^	LPP	150	200	300	400
Laser polishing speed	mm/s	LPS	500	600	800	1000
Laser polishing times	-	LPT	1	2	3	4

**Table 3:** The designed L_16_(4^3^) orthogonal experiments.

No.	A	B	C	LPPD (kW/cm^2^)	LPS (mm/s)	LPT
1	1	1	1	3900	500	1
2	1	2	2	3900	600	2
3	1	3	3	3900	800	3
4	1	4	4	3900	1000	4
5	2	1	2	5200	500	2
6	2	2	1	5200	600	1
7	2	3	4	5200	800	4
8	2	4	3	5200	1000	3
9	3	1	3	7790	500	3
10	3	2	4	7790	600	4
11	3	3	1	7790	800	1
12	3	4	2	7790	1000	2
13	4	1	4	10,400	500	4
14	4	2	3	10,400	600	3
15	4	3	2	10,400	800	2
16	4	4	1	10,400	1000	1

**Table 4:** Physical performance parameters of Ti6Al4V [21].

Property	Symbol	Unit	Value
Solid-phase specific heat capacity	$Cp_{s}$	J/(kg⋅K)	670
Liquid-phase specific heat capacity	$Cp_{l}$	J/(kg⋅K)	831
Solid-phase dynamic viscosity	$\mu_{s}$	Pa⋅s	100,000
Liquid-phase dynamic viscosity	$\mu_{l}$	Pa⋅s	0.005
Solid-phase density	$\rho_{s}$	${\mathrm{kg}/m}^{3}$	4420
Liquid-phase density	$\rho_{l}$	${\mathrm{kg}/m}^{3}$	4000
Solid-phase thermal conductivity	$k_{s}$	W/(m⋅K)	21
Liquid-phase thermal conductivity	$k_{l}$	W/(m⋅K)	29
Solid-phase temperature	$T_{s}$	K	1877
Liquid-phase temperature	$T_{l}$	K	1923
Melting temperature	$T_{m}$	K	1900
Evaporation temperature	$T_{v}$	K	3315
Transition temperature	∇T	K	23
Thermal expansion coefficient	β	K^−1^	1.1 × 10^−5^
Mushy zone constant	$A_{m}$	-	1 × 10^6^
Latent heat of fusion	$L_{m}$	J/kg	2.86 × 10^5^
Heat convection coefficient	$h_{c}$	W/(m⋅K)	10
Emissivity	ε	-	0.6
Stefan–Boltzmann constant	σ	$W/(m^{2}\cdot K^{4})$	5.67 × 10^−8^
Surface tension coefficient	γ	N/m	1.588
Surface tension temperature coefficient	$\frac{\sigma \gamma }{\sigma T}$	N/(m⋅K)	2.8 × 10^−4^
Inverse diffusion coefficient	$\beta_{r}$	-	0.18
Atmospheric pressure	$P_{0}$	Pa	1.01 × 10^5^
Latent heat of vaporization	$L_{v}$	J/kg	9.83 × 10^6^
Atomic mass	*m*	μ	45.9
Boltzmann constant	$K_{B}$	J/K	1.38 × 10^−24^

**Table 5:** Application of boundary conditions.

Boundary Condition	Applied Region
Heat flux	1
Heat convection	1, 2, 4
Thermal radiation	1, 2, 4
Adiabatic boundary	3
Capillary force	1
Thermocapillary force	1
No-slip boundary	2, 3, 4

**Table 6:** Experimental results of the L16(4^3^) orthogonal experiments.

No.	LPPD (kW/cm^2^)	LPS (mm/s)	LLT	Sa (μm)
1	3900	500	1	2.74
2	3900	600	2	1.69
3	3900	800	3	2.55
4	3900	1000	4	3.68
5	5200	500	2	2.46
6	5200	600	1	1.96
7	5200	800	4	1.85
8	5200	1000	3	2.81
9	7790	500	3	1.63
10	7790	600	4	2.75
11	7790	800	1	1.48
12	7790	1000	2	2.70
13	10,400	500	4	1.39
14	10,400	600	3	1.54
15	10,400	800	2	1.03
16	10,400	1000	1	1.48

**Table 7:** Range analysis table.

	LPPD (kW/cm^2^)	LPS (mm/s)	LPT
k1	3.55	2.74	2.55
k2	3.03	2.65	2.63
k3	2.85	2.30	2.84
k4	1.81	3.56	3.22
Optimal Level	4	3	1
R	1.74	1.26	0.67
Order of range	R_1_ > R_2_ > R_3_		

## Data Availability

The original contributions presented in this study are included in the article. Further inquiries can be directed to the corresponding author.
